# BRCAness as a Biomarker for Predicting Prognosis and Response to Anthracycline-Based Adjuvant Chemotherapy for Patients with Triple-Negative Breast Cancer

**DOI:** 10.1371/journal.pone.0167016

**Published:** 2016-12-15

**Authors:** Hitomi Mori, Makoto Kubo, Reiki Nishimura, Tomofumi Osako, Nobuyuki Arima, Yasuhiro Okumura, Masayuki Okido, Mai Yamada, Masaya Kai, Junji Kishimoto, Tetsuyuki Miyazaki, Yoshinao Oda, Takao Otsuka, Masafumi Nakamura

**Affiliations:** 1 Department of Surgery and Oncology, Graduate School of Medical Sciences, Kyushu University, Fukuoka, Japan; 2 Breast Center, Kumamoto Shinto General Hospital, Kumamoto, Japan; 3 Department of Pathology, Kumamoto Shinto General Hospital, Kumamoto, Japan; 4 Department of Breast and Endocrine Surgery, Kumamoto City Hospital, Kumamoto, Japan; 5 Department of Surgery, Hamanomachi Hospital, Fukuoka, Japan; 6 Department of Research and Development of Next Generation Medicine, Faculty of Medical Sciences, Kyushu University, Fukuoka, Japan; 7 Department of Anatomic Pathology, Graduate School of Medical Sciences, Kyushu University, Fukuoka, Japan; University of South Alabama Mitchell Cancer Institute, UNITED STATES

## Abstract

**Background:**

Triple-negative breast cancer (TNBC) is a heterogeneous tumor that encompasses many different subclasses of the disease. In this study, we assessed BRCAness, defined as the shared characteristics between sporadic and *BRCA1*-mutated tumors, in a large cohort of TNBC cases.

**Methods:**

The BRCAness of 262 patients with primary TNBCs resected between January 2004 and December 2014 was determined through the isolation of DNA from tumor tissue. Classification of BRCAness was performed using multiple ligation-dependent probe amplification (MLPA). The tumor subtypes were determined immunohistochemically using resected specimens.

**Results:**

Of the 262 TNBCs, the results of the MLPA assays showed that 174 (66.4%) tumors had BRCAness. Patients with BRCAness tumors were younger than patients with non-BRCAness tumors (*P* = 0.003). There was no significant difference between the two groups regarding their pathological stages. The BRCAness group had a significantly shorter recurrence-free survival (RFS) compared with the non-BRCAness group (*P* = 0.04) and had a shorter overall survival (OS) although this did not reach statistical significance. Adjuvant treatments with anthracycline-based regimens provided significantly greater benefits to the BRCAness group (*P* = 0.003 for RFS, and *P* = 0.03 for OS). Multivariate Cox proportional hazard model analysis showed that BRCAness was an independent negative prognostic factor, and the anthracycline-based adjuvant chemotherapy was an independent positive prognostic factor for both RFS and OS in TNBC.

**Conclusions:**

The 66.4% patients of TNBCs showed BRCAness. BRCAness is essential as a biomarker in the subclassification of TNBCs and might be of use for predicting their prognosis. Furthermore, this biomarker might be a predictive factor for the effectiveness of anthracycline-based adjuvant chemotherapy for patients with TNBCs.

## Introduction

Triple-negative breast cancer (TNBC) is a subclass of breast tumors that lack estrogen receptor (ER) and progesterone receptor (PgR) expression, as determined by immunohistochemistry (IHC). They also lack expression of human epidermal growth factor receptor type 2 (HER2), demonstrated by IHC and *in situ* hybridization. This specific subtype of TNBC accounts for 12%–17% of breast cancers [1], and cannot be treated with endocrine therapy or therapies targeted to HER2. As such, patients with TNBC have relatively poor outcomes. Adjuvant therapy is an important component in the treatment plan for patients with TNBC, as the peak time for distant recurrence from TNBC is 1–3 years after diagnosis [2]. TNBCs are heterogeneous and are composed of different intrinsic molecular subtypes, with basal-like (BL) tumors predominating [3]. Thus, classification of TNBC into subclasses is potential and needed to select treatments.

Over the years, basal-like breast cancer (BLBC) has become more commonly known as the major component of TNBC. Lehmann et al. published the list of 2,188 genes that classified TNBC into six subclasses (BL1, BL2, immunomodulatory [IM], mesenchymal [M], mesenchymal stem-like [MSL], and luminal androgen receptor [LAR]), using gene profiles obtained from 21 publicly available data sets [4]. Recently, Prat et al. reported that, among 412 TNBC, 78.6% were identified as basal-like, 7.8% as HER2-enriched, 6.6% as luminal, and 7.0% as normal-like [3]. In the process of developing the intrinsic subtypes, it has been suggested that *BRCA*-mutant tumors could be associated with basal-like profile [5]. Breast cancer of patients with germline *BRCA1* mutations are often TN and BL, and their *BRCA1* defects or deficiency may be involved in sporadic TNBC and BLBC [6]. From the current understanding of the biological functions of *BRCA*-mutant cancer [7], there appears to be a link between the *BRCA1* pathway and BLBC.

Tumors that share molecular features of *BRCA*-mutant tumors, which are referred to as ‘BRCAness’, may also respond to similar therapeutic strategies [8]. *BRCA*-mutant cancers have been shown to have increased sensitivity to DNA crosslinking agents such as platinum salts, because *BRCA*-mutant cancers have defects in DNA repair including homologous recombination [9–11]. Similarly, BRCAness tumors are highly sensitive to chemotherapy with DNA-damaging agents [12] or high-dose platinum [13, 14]. Although a major challenge is to find markers that clearly identify TNBC and BLBC, BRCAness may indicate essential approaches for therapeutic strategies.

To further identify the subclasses of TNBC, we examined the BRCAness of early TNBC in patients treated at three hospitals. Our goal was to explore the role of BRCAness as a prognostic factor of TNBC patients and as a predictive factor of conventional chemotherapy. We searched for possible correlations with their clinicopathological features including BRCAness and patient survival.

## Materials and Methods

### Patients

This study included 262 patients with primary TNBC who underwent resection without neoadjuvant chemotherapy at Kyushu University Hospital (Fukuoka, Japan), Hamanomachi Hospital (Fukuoka, Japan), or Kumamoto City Hospital (Kumamoto, Japan) between January 2004 and December 2014. The study conformed to the principles of the Declaration of Helsinki and was approved by the Institutional Review Board (IRB) of Kyushu University Hospital (No. 27–102). Prior to their operations, participants comprehensively provided their written consent stating that the tissue samples from resected specimen may be used for various research. Once the IRB approved this study, all details were made available on the Kyushu University Hospital website instead of renewing informed consent. All patients have the option to confirm ongoing studies and may choose to opt out of consent at any time. The IRB approved this consent procedure. The patients received adjuvant treatment according to the NCCN Guidelines for treatment of breast cancer by the National Comprehensive Cancer Network (http://www.nccn.org/professionals/physician_gls/f_guidelines.asp#breast), the Clinical Practice Guideline of Breast Cancer by the Japanese Breast Cancer Society (http://jbcs.xsrv.jp/guidline/, in Japanese), and the recommendations of the St. Gallen International Breast Cancer Conference [15–18]. Anthracycline-based adjuvant chemotherapies were AC (doxorubicin [60mg/m^2^] and cyclophosphamide [600mg/m^2^] every 3 weeks), EC (epirubicin [90mg/m^2^] and cyclophosphamide [600mg/m^2^] every 3 weeks), or FEC (5-fluorouracil [500mg/m^2^], epirubicin [100mg/m^2^], and cyclophosphamide [500mg/m^2^] every 3 weeks). Non-anthracycline-based adjuvant chemotherapies were TC (docetaxel [75mg/m^2^] and cyclophosphamide [600mg/m^2^] every 3 weeks), DTX (docetaxel [75mg/m^2^] every 3 weeks), CMF (cyclophosphamide [100mg/m^2^], methotrexate [40mg/m^2^], and 5-fluorouracil [600mg/m^2^] every 4 weeks), and others (S1 Table).

### Immunohistochemistry

Elucidation of tumor subtypes was undertaken by IHC on surgically resected tissue. Classification of ER- or PgR-positive tissue was defined as ≥ 1% of tumor cells staining positive for ER or PgR. Cancer specimens were defined as HER2-positive when the HER2 IHC staining was scored as 3+ according to the standard criteria [19, 20] or by fluorescence spectroscopy using *in situ* hybridization, which showed HER2 gene amplification. The epidermal growth factor receptor (EGFR) primary antibody (monoclonal mouse, clone DAK-H1-WT, Dako, Glostrup, Denmark) was used with a Ventana Discovery XT automated stainer (Ventana Medical Systems, AZ, USA) as per the manufacturer’s protocol with proprietary reagents. Briefly, slides were deparaffinized on the automated system with EZ Prep solution. A heat-induced antigen retrieval method was used in standard Cell Conditioning 1 (CC1) with an incubation temperature of 95°C. The primary antibody was used at a 1:50 dilution and incubated for 32 min. The secondary antibody was included with the I-VIEW DAB universal kit detection system (Ventana Medical Systems). Slides were counterstained with hematoxylin and then a bluing reagent was used for post-counterstaining. Cytokeratin 5/6 (CK5/6) primary antibody (monoclonal mouse, clone D5/16 B4, Dako) was used following the same staining standard CC1 protocol at a 1:100 dilution. The BL phenotype was defined as being positive for EGFR and/or CK5/6 [21] (S1 Fig).

### MLPA method

Surgical specimens were used for multiple ligation-dependent probe amplification (MLPA) analysis. DNA was isolated from formalin-fixed paraffin-embedded (FFPE) tumor tissues using a QIAamp DNA FFPE tissue kit (Qiagen, Hilden, Germany). Classification of BRCAness subtypes was performed using MLPA with a P376 BRCA1ness probemix (MRC-Holland, Amsterdam, the Netherlands), as previously reported [12]. MLPA was undertaken to determine the relative copy number of various DNA sequences, and was performed according to the manufacturer’s instructions [22]. The MLPA probe mix contained 38 target probes, which covered the most important genomic regions of the *BRCA1*-like classifier based on specific aberrations of *BRCA1*-mutated breast cancer compared with sporadic tumors by array comparative genomic hybridization (aCGH), and eight control reference probes [12]. Data analysis was performed using the Coffalyser.NET software (MRC-Holland). The relative copy number ratio of each sample was compared using Human Genomic DNA (Promega, Madison, WI, USA) as a reference sample. BRCAness scores were calculated with the relative copy number ratios of various DNA sequences. The relative copy number ratios from Coffalyser.NET for all the target-specific probes were used in the prediction analysis for the microarrays (PAM). The training set generated by MRC-Holland with P376-B2 Lot 0911 was used for the PAM. Each sample was analyzed twice and the average score was used for this analysis. The BRCAness analysis was performed by researchers completely blinded to the clinical information. A sample with a BRCAness score of ≥0.5 was classified as BRCAness. If the score was <0.5, the sample was classified as being non-BRCAness [23].

### Statistics

Logistic regression was used to compare the continuous variables and χ^2^ test was used to assess the categorical variables between the BRCAness and non-BRCAness groups. The survival endpoints evaluated were RFS and OS. RFS was defined as the time from surgery to recurrence, including both local relapse and metastatic disease. OS was defined as the time from surgery until the date of death from any cause. Survival curves were generated using the Kaplan–Meier method and were compared with the log-rank test. Hazard ratios were calculated using a Cox proportional hazards regression. A *P* value of <0.05 was considered statistically significant. Statistical analysis was carried out using JMP® 11 (SAS Institute Inc., Cary, NC, USA).

## Results

### MLPA assay and clinicopathological features

Of the 262 TNBCs, 174 (66.4%) tumors had BRCAness as shown by the MLPA assay. Patients with BRCAness tumors were younger than the patients with non-BRCAness tumors (*P* = 0.003; Table 1). Nuclear grade and Ki-67 index of BRCAness tumors were higher when compared with non-BRCAness tumors (*P* < 0.0001 and *P* = 0.002, respectively), although there was no significant difference between the two groups regarding tumor size, nodal status, and pathological stage (Table 1). The BRCAness tumors included the BL phenotype more than the non-BRCAness tumors (*P* = 0.04; Table 1). The ratio value of four *BRCA* probes, *BRCA1*-exon20, *BRCA1*-exon2, *BRCA2*-exon5, and *BRCA2*-exon11, which were included in the BRCA1ness MLPA kit, on BRCAness tumors was significantly lower compared with non-BRCAness tumors (*P* < 0.0001; S2 Fig).

**Table 1:** Patients and tumor characteristics (*n* = 262).

	BRCAness		Non-BRCAness		
	(*n* = 174)		(*n* = 88)		*P* value
Age at diagnosis					
Mean (range)	58.6	(30–87)	63.6	(31–89)	**0.003**^a^
Time to last follow-up (months)					
Mean (range)	54.4	(2–124)	52.7	(2–139)	0.71^a^
Tumor size					
pT1a/b (≤1cm)	14	(8.1%)	12	(13.6%)	0.42^b^
pT1c (>1cm, ≤2cm)	92	(52.9%)	39	(44.3%)	
pT2 (>2cm, ≤5cm)	63	(36.2%)	34	(38.6%)	
pT3 (>5cm)	5	(2.9%)	3	(3.4%)	
Nodal status					
pN0	117	(67.2%)	61	(69.3%)	0.67^b^
pN1 (1−3)	42	(24.1%)	19	(21.6%)	
pN2 (4−9)	10	(5.8%)	4	(4.6%)	
pN3 (≥10)	5	(2.9%)	3	(3.4%)	
Unknown	0		1	(1.1%)	
Pathological stage					
I	77	(44.3%)	39	(44.3%)	0.99^b^
II	82	(47.1%)	41	(6.68%)	
III	15	(8.6%)	8	(9.1%)	
Nuclear grade					
1+2	35	(20.1%)	49	(55.7%)	**<0.0001**^b^
3	135	(77.6%)	35	(39.8%)	
Unknown	4	(2.3%)	4	(4.6%)	
Ki-67					
≤20%	12	(6.9%)	19	(21.6%)	**0.002**^b^
20%<	136	(78.2%)	54	(61.4%)	
Unknown	26	(14.9%)	15	(17.1%)	
Basal-like status					
Basal-like	159	(91.4%)	73	(83.0%)	**0.04**^b^
Non-basal-like	15	(8.6%)	15	(17.0%)	
Adjuvant chemotherapy					
Anthracycline-based regimens	115	(66.1%)	44	(50.0%)	0.07^b^
Non-anthracycline-based regimens	11	(6.3%)	9	(10.2%)	
No treatment	47	(27.0%)	35	(39.8%)	
Unknouwn	1	(0.6%)	0		

^a^ Logistic regression

^b^ Pearson's χ^2^ test.

### Patient survival

**Fig 1:**
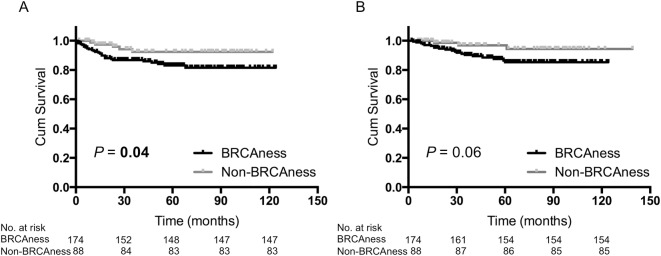
Kaplan–Meier analysis of patients with TNBC (*n* = 262). (A) Recurrence-free survival of BRCAness tumors versus non-BRCAness tumors. (B) Overall survival of BRCAness tumors versus non-BRCAness tumors.

The median follow-up was 54 months (range 2–139 months) in this cohort. The BRCAness group had a significantly shorter recurrence-free survival (RFS) compared with the non-BRCAness group (*P* = 0.04; Fig 1A), and had a shorter overall survival (OS) although this did not reach statistical significance (*P* = 0.06; Fig 1B).

**Fig 2:**
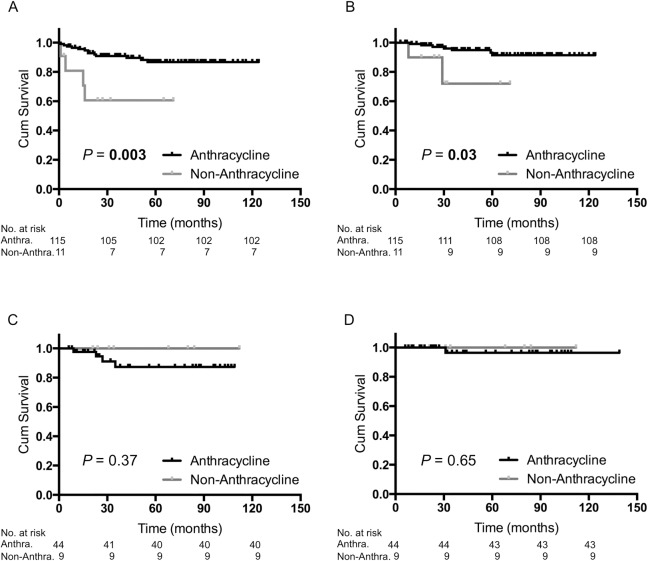
Kaplan–Meier analysis of patients who received anthracycline-based adjuvant chemotherapy versus non-anthracycline-based adjuvant chemotherapy. (A) Recurrence-free survival (RFS) of BRCAness tumors (*n* = 126). (B) Overall survival (OS) of BRCAness tumors (*n* = 126). (C) RFS of non-BRCAness tumors (*n* = 53). (D) OS of non-BRCAness tumors (*n* = 53). Anthracycline or Anthra, anthracycline-based adjuvant chemotherapy; Non-Anthracycline or Non-Anthra, non-anthracycline-based adjuvant chemotherapy.

Among the 174 patients with BRCAness tumors, 115 (66.1%) patients received anthracycline-based adjuvant chemotherapy, 11 (6.3%) patients received non-anthracycline-based adjuvant chemotherapy, 47 (27.0%) patients received no treatment, and there was no information available for one (0.6%) patient (Table 1). Anthracycline-based regimens provided significantly greater benefit to patients with BRCAness tumors (*P* = 0.003 for RFS, Fig 2A; and *P* = 0.03 for OS, Fig 2B). In contrast, among the 88 patients with non-BRCAness tumors, 44 (50.0%) patients received anthracycline-based adjuvant chemotherapy, nine (10.2%) patients received non-anthracycline-based adjuvant chemotherapy, and 35 (39.8%) patients received no treatment (Table 1). Regardless of whether an anthracycline was used for patients with non-BRCAness tumors, there was no significant difference between these two groups regarding their RFS and OS (Fig 2C and 2D).

**Fig 3:**
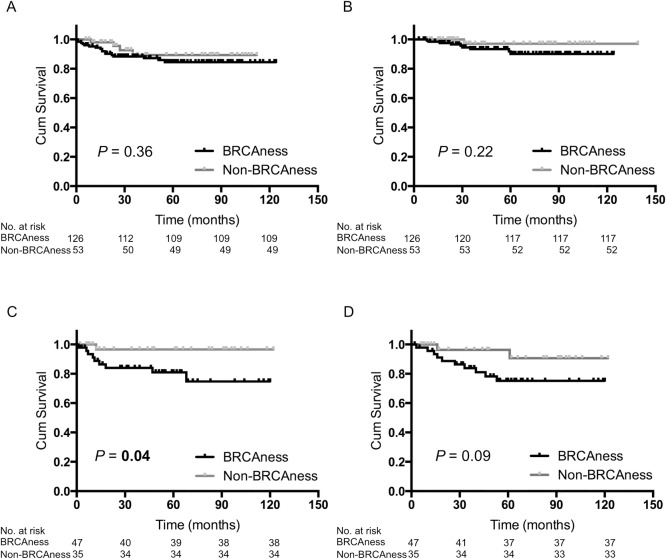
Kaplan–Meier analysis of patients with BRCAness tumors versus non-BRCAness tumors. (A) RFS of patients who received adjuvant chemotherapy (*n* = 179). (B) OS of patients who received adjuvant chemotherapy (*n* = 179). (C) Recurrence-free survival (RFS) of patients who received no treatment (*n* = 82). (D) Overall survival (OS) of patients who received no treatment (*n* = 82).

When we divided TNBC patients into two groups—those who received adjuvant chemotherapy and those who received no treatment—among the 179 patients who received adjuvant chemotherapy, 126 (70.4%) tumors had BRCAness (Table 1). There was no significant difference for prognosis between the patients who had BRCAness tumors and who had non-BRCAness tumors in the cases of the adjuvant chemotherapy group (Fig 3A and 3B). In contrast, among the 82 patients who received no treatment, 47 (57.3%) tumors had BRCAness (Table 1). In the cases of the no treatment group, the patients with BRCAness tumors had a significantly shorter RFS compared to the patients with non-BRCAness tumors (*P* = 0.04; Fig 3C) and had a shorter OS, although this did not reach statistical significance (*P* = 0.09; Fig 3D).

### Univariate and multivariate survival analysis

The univariate analysis revealed that tumor size (>2cm), lymph node involvement, pathological stage III, and BRCAness were significantly related to poorer RFS and OS (Table 2A). The factors of tumor size and nodal status were excluded from the multivariate analysis because they were included as part of the pathological stage. The age at diagnosis, nuclear grade, Ki-67 index, and BL status were also excluded from the multivariate analysis by the back elimination method. The multivariate analysis demonstrated that pathological stage III and BRCAness proved to be independent negative prognostic factors for both RFS and OS (Table 2B). Meanwhile, the anthracycline-based adjuvant chemotherapy proved to be an independent positive prognostic factor against the non-anthracycline-based adjuvant chemotherapy for both RFS and OS (Table 2B).

**Table 2:** Cox proportional hazards model for recurrence-free survival and overall survival (*n* = 262).

A. Univariate analysis							
		Recurrence-free survival			Overall survival		
Variable		HR	95% CI	*P* value	HR	95% CI	*P* value
Age	(50< vs. ≤50)	1.8	0.8–5.4	0.19	2.2	0.8–9.4	0.16
Tumor size	(2cm< vs. ≤2cm)	3.7	1.8–8.3	**0.0003**	3.9	1.7–10.2	**0.002**
Nodal status	(Positive vs. Negative)	4.4	2.1–9.4	**<0.0001**	4.3	1.9–10.7	**0.0006**
pStage	(III vs. I and II)	6.8	3.1–14.1	**<0.0001**	9.5	4.0–21.9	**<0.0001**
Nuclear grade	(3 vs. 1 and 2)	1.1	0.5–2.4	0.87	1.0	0.4–2.4	0.93
Ki-67	(20%< vs. ≤20%)	1.2	0.4–5.1	0.76	1.0	0.3–4.1	0.96
Basal-like status	(Basal-like vs. Non-basal-like)	0.9	0.3–2.9	0.78	1.3	0.4–8.1	0.72
BRCAness status	(BRCAness vs. Non-BRCAness)	2.6	1.1–7.8	**0.03**	3.1	1.1–13.2	**0.04**
Chemotherapy	(Anthra vs. Non-anthra)	0.4	0.2–1.6	0.19	0.4	0.1–2.6	0.29
	(Anthra vs. No Treatment)	0.8	0.4–1.9	0.64	0.3	0.1–0.8	**0.02**
B. Multivariate analysis							
		Recurrence-free survival			Overall survival		
Variable		HR	95% CI	*P* value	HR	95% CI	*P* value
pStage	(III vs. I and II)	9.5	4.1–21.1	**<0.0001**	21.3	7.7–60.1	**<0.0001**
BRCAness status	(BRCAness vs. Non-BRCAness)	3.3	1.3–10.1	**0.008**	6.3	1.9–29.0	**0.002**
Chemotherapy	(Anthra vs. Non-anthra)	0.2	0.1–0.7	**0.02**	0.1	0.02–0.7	**0.03**
	(Anthra vs. No Treatment)	0.6	0.2–1.3	0.17	0.2	0.1–0.4	**0.0002**

HR, hazard ratio; CI, confidence interval; vs., versus; Anthra, Anthracycline-based adjuvant chemotherapy; non-Anthra, Non-anthracycline-based adjuvant chemotherapy.

## Discussion

The aim of this study was to determine whether BRCAness was capable of being prognostic and whether patients identified as having TNBC tumors benefited from conventional chemotherapy. For this purpose, we retrospectively analyzed a relatively large sample of TNBC patients treated at three hospitals.

We used the MLPA assay [12] to examine whether the tumors had BRCAness, which was derived from the genomic profiles of *BRCA1*-mutated breast cancers. The MLPA assay is a rapid, cost-efficient, and suitable test of FFPE tissue-derived DNA, and therefore is potentially useful for routine clinical application [12]. Our results from the MLPA assay revealed that 66.4% of the 262 TNBC had BRCAness. BRCAness accounted for 11%–14% of the sporadic breast cancers [8] and 66%–69% of the TNBCs [24]. Furthermore, 16% of all TNBC patients and 36% of TNBC patients who were under 40 years old had *BRCA1* mutations [25]. The patients with BRCAness tumors were younger than those with non-BRCAness tumors (*P* < 0.0001). These findings confirm the results of Oonk et al. [26] and Lips et al. [24]. The nuclear grades and Ki-67 indexes of the BRCAness tumors were higher than those of the non-BRCAness tumors (*P* < 0.0001 and *P* = 0.002, respectively), and BRCAness tumors included more BL tumors compared to non-BRCAness tumors (*P* = 0.04). However, age at diagnosis, nuclear grade, Ki-67 index, and BL status were not prognostic factors as determined by univariate analysis. Meanwhile, the pathological stage by TNM classification was a prognostic factor as determined by univariate and multivariate analyses.

Most importantly, this study showed a difference in survival between BRCAness and non-BRCAness tumors from examination of the 262 TNBCs. Furthermore, in the cases who did not receive adjuvant chemotherapy, the patients with BRCAness tumors had a significant shorter RFS compared to the patients with non-BRCAness tumors. The natural progression of BRCAness tumors might have a higher recurrence risk than that of non-BRCAness tumors. However, in the cases that received adjuvant chemotherapy, there was no significant difference between the patients who had BRCAness tumors and non-BRCAness tumors regarding both RFS and OS. Even if tumor size was under 1cm and lymph node involvement was negative, patients with BRCAness tumors should be strongly considered to receive adjuvant chemotherapy, especially anthracycline-based regimens. According to the results of the multivariate analysis, pathological stage III, BRCAness, and treatment with anthracycline-based chemotherapy were significantly associated with RFS and OS, which were independent of other factors. Prat et al. found that proliferation genomic signatures predicted response and improved survival after chemotherapy, but only for tumors with a BL phenotype. This result suggested that future clinical trials should focus on this phenotype and should be considered for determining if patients have BLBC [27]. Our data showed that 232 (88.5%) tumors of 262 TNBCs had the BL phenotype by IHC of EGFR and CK5/6.

The most common chemotherapeutic approach to advanced TNBC is based on a treatment regimen that includes an anthracycline and a taxane. In this study, treatment with an anthracycline-based regimen for BRCAness was significantly more effective with regards to both RFS and OS compared with the other treatments (*P* = 0.003, and *P* = 0.03, respectively). Neoadjuvant studies indicate that TNBCs and BLBCs respond well to anthracycline-based therapy (pathological complete response [pCR] = 29%, [28]) or anthracycline- and taxane-based therapy (pCR = 45%, [29]). Unfortunately, despite the higher response rates, relapse rates are higher in patients who did not achieve a pCR, which resulted in worse OS for the BLBC and HER2-enriched groups compared with patients with luminal tumors [28]. Meta-analyses by the Early Breast Cancer Trialists’ Collaborative Group [30] showed that in trials where a taxane was added to an anthracycline-based control regimen, breast cancer mortality was reduced significantly in ER-poor groups (relative risk [RR] 0.86, standard error [SE] 0.04). Moreover, in trials that compared standard anthracycline-based treatment with no chemotherapy, breast cancer mortality was reduced significantly in ER-poor groups (RR 0.86, SE 0.07). In contrast, survival outcomes of standard 4AC and standard CMF appeared equivalent. Conforti et al. also reported from two randomized trials that ER expression can predict efficacy of adjuvant anthracycline-based chemotherapy (FEC50) when compared with no treatment [31]. Miyoshi et al. suggested that both TOP2A and BRCA1 influence sensitivity to anthracyclines, and tumors with a phenotype, such as TOP2A-positive or BRCA-negative, seem to constitute a highly sensitive group [32].

The ratio value of four *BRCA* probes, *BRCA1*-exon20, *BRCA1*-exon2, *BRCA2*-exon5, and *BRCA2*-exon11, on BRCAness tumors was significantly lower compared with non-BRCAness tumors (*P* < 0.0001). A study from Rakha et al. [33] showed that sporadic BLBCs could have reduced *BRCA1* protein expression, which is possibly caused by reduced transcription as a result of loss of heterozygosity and/or *BRCA1* promoter methylation. Recently, homologous recombination deficiency (HRD) score, based on an unweighted sum of loss of heterozygosity, telomeric allelic imbalance, and large-scale state transitions measured in tumors with *BRCA1/2* deficiencies, can predict the likelihood of response to platinum-based neoadjuvant chemotherapy in patients with TNBC [34–36]. At the same time, Isakoff et al. [37] reported that, in metastatic setting trial TBCRC009, a measure of DNA repair function may identify TNBC patients without germline *BRCA1/2* mutations who benefit from platinum-based regimens.

Vollebergh et al. reported that an aCGH classifier based on *BRCA1*-mutated breast cancer might be predictive for selective high-dose platinum-based chemotherapy, a DNA double-strand break-inducing regimen [13]. These include platinum-based drugs, such as cisplatin or carboplatin, mitomycin C, and topoisomerase poisons, such as camptothecin, or alkylating agents. The use of cisplatin or carboplatin to treat TNBCs is currently being assessed in clinical trials on the basis that dysfunction of *BRCA1* and its pathway is associated with a specific DNA-repair defect. Byrski et al. reported that pCR was observed in 65 out of 107 patients (61%), and that platinum-based chemotherapy is effective in a high proportion of patients with *BRCA1*-associated breast cancer [38], and potentially also for patients with TNBC tumors, including *BRCA1/2* non-mutated tumors [39].

Poly (adenosine diphosphate-ribose) polymerase (PARP) enzymes are critical to cell proliferation and are differentially upregulated in many cancers including TNBC and *BRCA1/2*-associated tumors [40]. Olaparib is an oral PARP inhibitor that was shown in a phase II trial to be useful against *BRCA1/2*-associated advanced breast cancers [41]. The results of this study provide an important proof-of-concept for PARP inhibition of *BRCA*-deficient breast cancers [42], and BRCAness may be a biomarker for selection of PARP inhibitor treatment.

## Conclusions

Our data suggests that BRCAness might be a prognostic marker for TNBCs as well as a predictive tool in determining the benefit of anthracycline-based chemotherapy. This is the first study of Japanese patients with TNBC where these markers were evaluated with long-term survival outcomes. However, further studies are needed to clarify the molecular mechanisms involved in the regulation of each subclass in TNBC.

## Supporting Information

S1 Fig Basal-like phenotype of TNBC. (A) Hematoxylin and eosin stain. (B) Immunohistochemistry of EGFR. (C) Immunohistochemistry of CK5/6.
(TIF)

S2 Fig The ratio value of four BRCA probes of MLPA. (A) *BRCA1*-exon2. (B) *BRCA1*-exon20. (C) *BRCA2*-exon5. (D) *BRCA2*-exon11.
(TIF)

S1 Table Chemotherapy regimens (*n* = 179). (DOCX)
