# Supplementary figures and images for: BRCAness as a Biomarker for Predicting Prognosis and Response to Anthracycline-Based Adjuvant Chemotherapy for Patients with Triple-Negative Breast Cancer
Source: PLoS One. 2016 Dec 15;11(12):e0167016. doi: 10.1371/journal.pone.0167016 (PMC5158199; doi:10.1371/journal.pone.0167016)

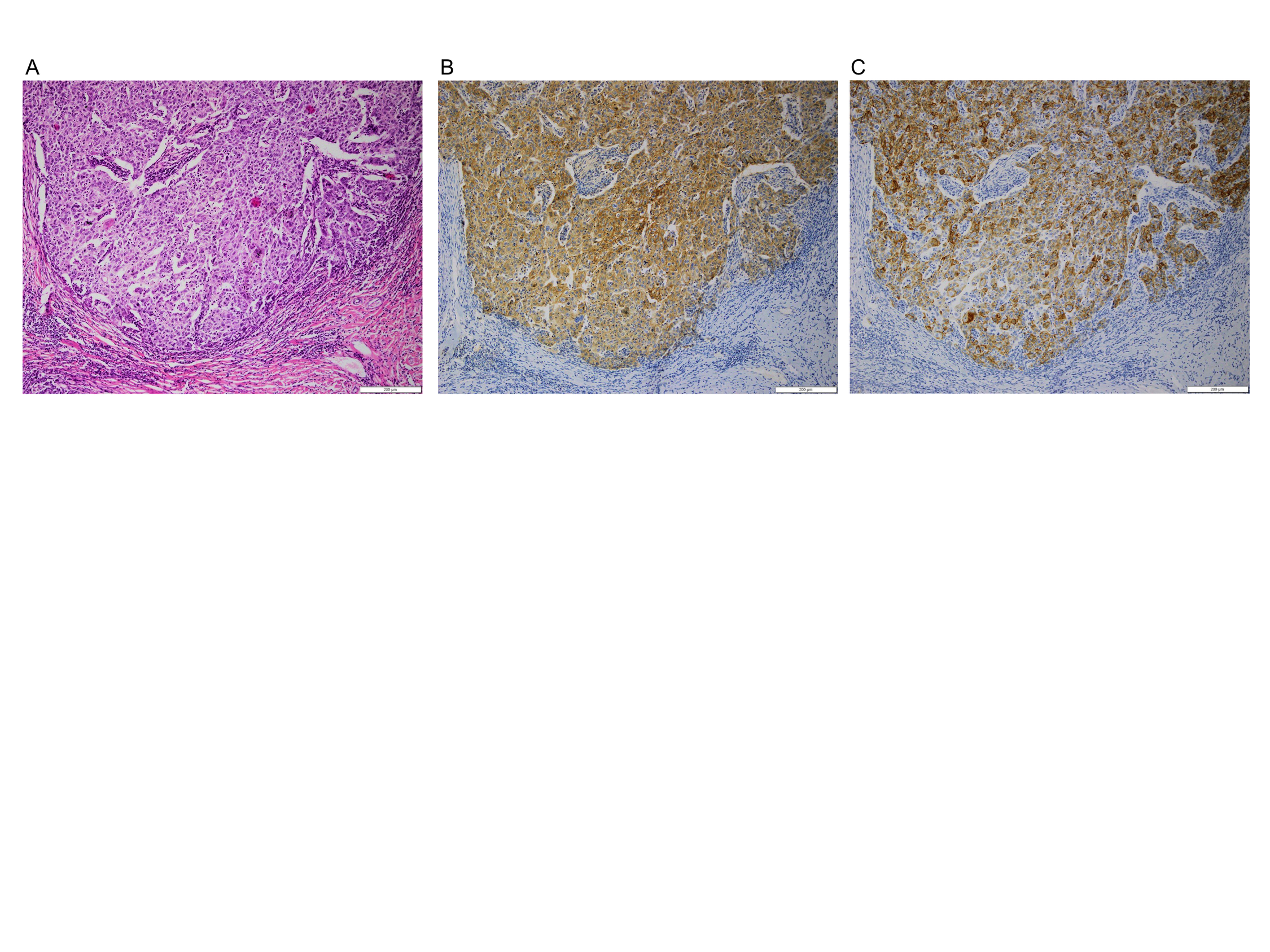

Supplement: S1 Fig — (A) Hematoxylin and eosin stain. (B) Immunohistochemistry of EGFR. (C) Immunohistochemistry of CK5/6. (TIF) [file pone.0167016.s001.tif]

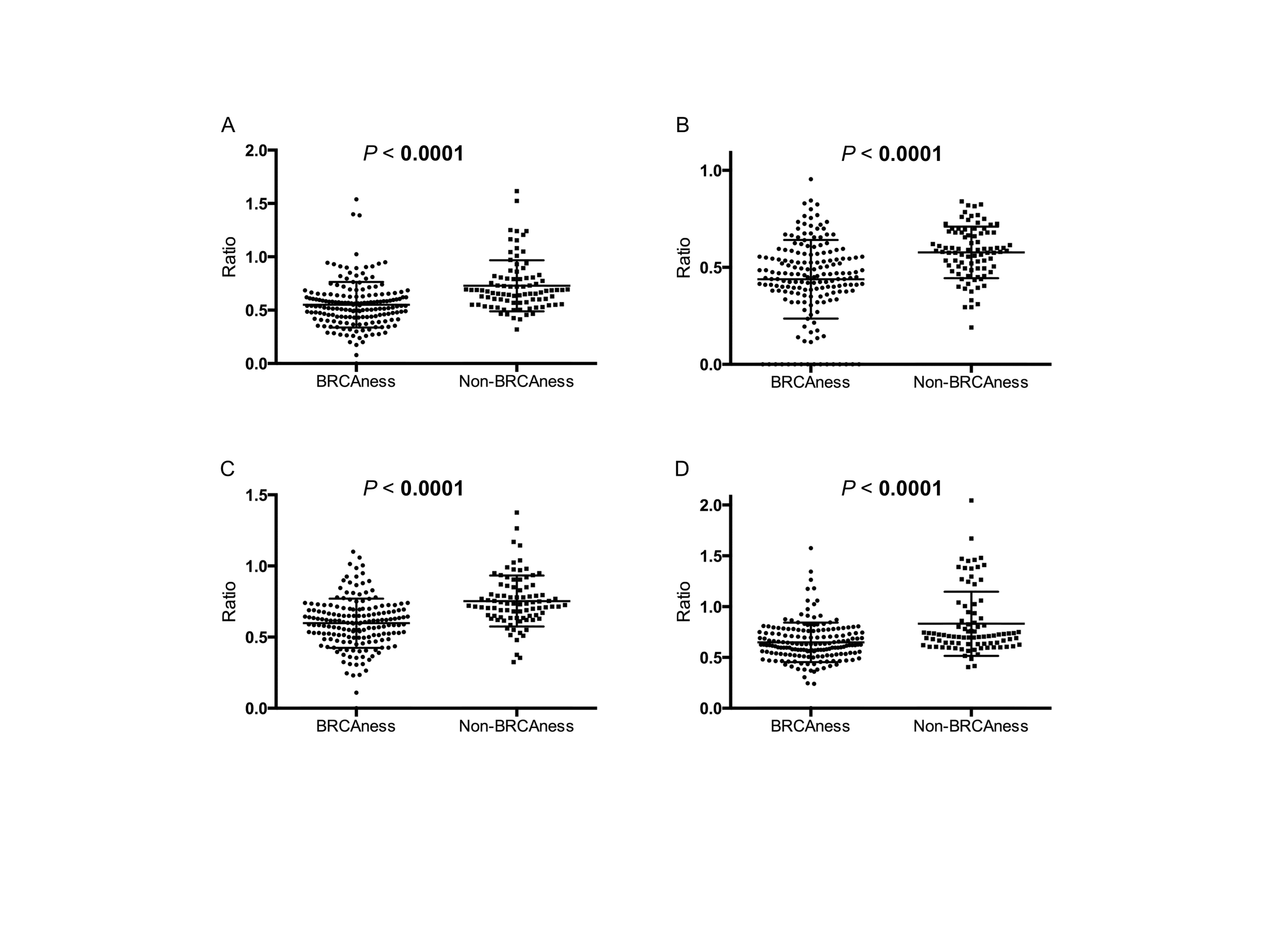

Supplement: S2 Fig — (A) BRCA1-exon2. (B) BRCA1-exon20. (C) BRCA2-exon5. (D) BRCA2-exon11. (TIF) [file pone.0167016.s002.tif]
