# Supplementary material for: BRCAness as a Biomarker for Predicting Prognosis and Response to Anthracycline-Based Adjuvant Chemotherapy for Patients with Triple-Negative Breast Cancer
Source: PLoS One. 2016 Dec 15;11(12):e0167016. doi: 10.1371/journal.pone.0167016 (PMC5158199; doi:10.1371/journal.pone.0167016)
Supplement: S1 Table — (DOCX) [file pone.0167016.s003.docx]

**S1 Table. Chemotherapy regimens (*n* = 179).**

|  | BRCAness | | Non-BRCAness | |  |
| --- | --- | --- | --- | --- | --- |
|  | (*n* = 126) | | (*n* = 53) | | *P* value |
| Anthracycline-based regimens |  |  |  |  |  |
| AC, EC, FEC | 66 | (52.4%) | 27 | (50.9%) | 0.31^a^ |
| EC+PTX, FEC+DTX | 49 | (38.9%) | 17 | (32.1%) | |
| Non-anthracycline-based regimens |  |  |  |  |  |
| TC, DTX | 4 | (3.2%) | 5 | (9.4%) |  |
| CMF | 3 | (2.4%) | 3 | (5.7%) |  |
| Others | 4 | (3.2%) | 1 | (1.9%) |  |

^a^ Pearson's χ^2^ test; AC, doxorubicin, cyclophosphamide; EC, epirubicin, cyclophosphamide; FEC, 5-fluorouracil, epirubicin, cyclophosphamide; PTX, paclitaxel; DTX, docetaxel; TC, docetaxel, cyclophosohamide; CMF, cyclophosphamide, methotrexate, 5-fluorouracil.
